# Mitral valve annuloplasty versus replacement for severe ischemic mitral regurgitation

**DOI:** 10.1038/s41598-018-19909-7

**Published:** 2018-01-24

**Authors:** Baotong Li, Shanglin Chen, Hansong Sun, Jianping Xu, Yunhu Song, Wei Wang, Shuiyun Wang

**Affiliations:** State Key Laboratory of Cardiovascular Disease, Department of Adult Cardiac Surgery, Fuwai Hospital,National Center for Cardiovascular Disease, Chinese Academy of Medical Science, Peking Union Medical College, Beijing, China

## Abstract

Although practice guidelines recommend surgery for patients with severe chronic ischemic mitral regurgitation (CIMR), they do not specify whether to repair or replace the mitral valve. 436 consecutive patients with severe CIMR were eligible for inclusion in the study, of which 316 (72.5%) underwent mitral valve annuloplasty (MVA) whereas 120 (27.5%) received mitral valve replacement (MVR). At 59 months (interquartile range, 37–85 months) follow-up, though the left ventricle end-diastolic diameter was markedly larger (*P* = 0.019) in the MVA group than in the MVR group, no significant difference was observed in overall survival, freedom from cardiac death, or avoidance of major adverse cardiac or cerebrovascular events (MACCE). MVA provides better results in freedom from cardiac death in subgroups of age ≥65years and left ventricular ejection fraction (EF) ≥50% (*P* = 0.014 and *P* = 0.016, respectively), whereas MVR was associated with a lower risk of MACCE in subgroups of age <65years, EF <50% and left ventricular inferior basal wall motion abnormality (BWMA) (all *P* < 0.05). In conclusion, MVR is a suitable management of patients with severe CIMR, and it is more favorable to ventricular remodeling. The choice of MVA or MVR should depend on major high-risk clinical factors.

## Introduction

The optimal management of patients with severe CIMR, specifically the choice between mitral valve annuloplasty (MVA) and mitral valve replacement (MVR), has long been debated^1–6^. Recent studies have showed that, compared with MVR, although MVA is associated with lower early postoperative mortality^7,8^, it provides worse long-term mitral regurgitation correction with risks of adverse left ventricular remodeling, atrial fibrillation, and readmission^2^. To date, however, very limited evidence has been available on the long-term outcomes of MVA and MVR for patients with severe CIMR. Therefore, we designed a retrospective, long-term, propensity score (PS) matched analysis to evaluate the effectiveness of MVA versus MVR for severe CIMR.

## Results

### Patient characteristics

The baseline and procedural characteristics of study patients are illustrated in Table 1. Three kinds of complete symmetric rings were used in the present study, with the median size of 28 mm (interquartile range, 28–29 mm): Carpentier–Edwards Physio ring I (Edwards Lifesciences, Irvine, CA), Carpentier–Edwards Physio ring II (Edwards Lifesciences, Irvine, CA), Duran Ancore (Medtronic, Santa Ana, CA). There were seven types of prosthetic valves, with the median size of 27 mm (interquartile range, 27–29 mm). The rate of bioprosthesis was 45.8% (55/120). Three types of bioprostheses were used (n = 55): Carpentier-Edwards Perimount (Edwards Lifesciences, Irvine, CA), Mosaic (Medtronic, Santa Ana, CA) and Hancock II (Medtronic, Santa Ana, CA). Four types of mechanical valves were used (n = 65): On-X valve (On-X Life Technology, Austin, TX), Medtronic Open Pivot (Medtronic, Minneapolis, MN), CarboMedics Mechanical (Sorin-CarboMedics Inc, Italia, S.r.l), and St. Jude valve (St. Jude Medical, Minneapolis, MN). Mitral leaflets were preserved whenever possible (94/120, 78.3%), with posterior leaflet preservation in 58(48.3%) patients, posterior and partial anterior leaflet preservation in 8(6.7%) patients, and both leaflets preservation in 28(23.3%) patients.

**Table 1:** Baseline demographic and clinical characteristics of patients based on surgical procedures.

	Overall patient			Pairs matched by PS			
	MVA (n = 316)	MVR (n = 120)	P Value	MVA (n = 109)	MVR (n = 109)	P Value	Standardized difference
Age, year*	59.42 ± 8.51	61.49 ± 9.09	0.027	61.72 ± 7.95	60.83 ± 8.84	0.435	−0.098
Sex (male), n (%)*	256 (81.0)	91 (75.8)	0.231	82 (75.2)	85 (78.0)	0.631	0.064
Body surface area, m^2^*	1.78 ± 0.18	1.75 ± 0.16	0.035	1.75 ± 0.15	1.76 ± 0.15	0.631	0.063
Diabetes, n (%)*	75 (23.7)	22 (18.3)	0.226	17 (15.6)	22 (20.2)	0.377	0.118
Hypertension, n (%)*	163 (51.6)	66 (55.0)	0.523	64 (58.7)	60 (55.0)	0.584	−0.073
Hyperlipidemia, n (%)*	128 (40.5)	56 (46.7)	0.245	50 (45.9)	50 (45.9)	>0.999	0
COPD, n (%)*	22 (7.0)	7 (5.8)	0.673	6 (5.5)	6 (5.5)	>0.999	0
History of PCI, n (%)*	40 (12.7)	14 (11.7)	0.779	10 (9.2)	13 (11.9)	0.508	0.085
History of heart failure, n (%)*	166 (52.5)	64 (53.3)	0.881	59 (54.1)	56 (51.4)	0.684	−0.055
History of stroke, n (%)*	28 (8.9)	15 (12.5)	0.255	11 (10.1)	14 (12.8)	0.524	0.083
Ventricular arrhythmia, n (%)*	17 (5.4)	5 (4.2)	0.605	5 (4.8)	5 (4.8)	>0.999	0
Atrial fibrillation, n (%)*	39 (12.3)	12 (10.0)	0.497	8 (7.3)	5 (4.6)	0.391	−0.061
LV aneurysm, n (%)*	34 (10.8)	4 (3.3)	0.014	3 (2.8)	4 (3.7)	0.7	0.051
Unstable angina, n (%)*	61 (19.3%)	17 (14.2)	0.211	22 (20.2)	15 (13.8)	0.207	−0.183
NYHA functional class*	2.58 ± 0.59	2.61 ± 0.64	0.689	2.61 ± 0.62	2.56 ± 0.60	0.507	−0.086
Left main CAD, n (%)*	62 (19.6)	17 (14.2)	0.187	17 (15.6)	15 (13.8)	0.702	−0.052
EF*	51.50 ± 11.97	56.05 ± 10.15	<0.001	54.94 ± 10.42	56.11 ± 10.06	0.402	0.115
LVEDD*	58.74 ± 6.59	58.28 ± 6.12	0.513	58.04 ± 6.46	58.43 ± 6.25	0.647	0.065
LAD*	43.22 ± 6.08	43.94 ± 6.91	0.286	43.63 ± 6.60	43.91 ± 7.11	0.767	0.04
Grade of MR, n (%)*			<0.001			0.603	0.063
3+	289 (91.5%)	90 (75.0%)		90 (82.6%)	87 (79.8%)		
4+	27 (8.5%)	30 (25.0%)		19 (17.4%)	22 (20.2%)		
Pulmonary hypertension, n (%)	30 (9.5%)	24 (20.0%)	0.003	10 (9.2%)	19 (17.4%)	0.073	—
BWMA, n (%)*	181 (57.3)	68 (56.7)	0.908	60 (55.0)	61 (56.0)	0.892	0.018
CABG							
LIMA, n (%)*	271 (85.8)	100 (83.3)	0.525	91 (83.5)	90 (82.6)	0.857	−0.025
Radial artery, n (%)*	2 (0.6)	2 (1.7)	0.339	2 (1.8)	2 (1.8)	>0.999	0
Grafts/patient*	2.64 ± 0.84	2.59 ± 0.69	0.556	2.59 ± 0.88	2.58 ± 0.71	0.933	−0.013
Distal anastomoses/patient*	3.13 ± 1.12	2.91 ± 0.97	0.053	2.89 ± 1.09	2.91 ± 1.00	0.897	0.019
Concomitant procedure, n (%)							—
TAP*	24 (7.6)	25 (20.8)	<0.001	17 (15.6)	19 (17.4)	0.715	0.045
Modified maze procedure	2 (0.6)	0 (0.0)	—	0 (0.0)	0 (0.0)	—	
ACC time	109.59 ± 69.09	113.53 ± 36.47	0.554	98.08 ± 27.48	114.43 ± 37.13	<0.001	—
CPB time	154.72 ± 49.02	163.68 ± 57.03	0.104	144.43 ± 42.41	165.04 ± 58.14	0.003	—
Postoperative IABP, n (%)	18 (5.7)	5 (4.2)	0.523	5 (4.6)	4 (3.7)	0.734	—
Duration of intubation, hours; Median (IQR)	20 (15–30)	21 (16–37)	0.19	18 (15–30)	22 (17–39)	0.253	—
Duration of ICU, hours; Median (IQR)	69 (40–91)	85 (43–129)	0.061	64 (40–81)	84 (44–114)	0.036	—

MVA: mitral valve annuloplasty, MVR: mitral valve replacement, PS: propensity score, COPD: Chronic obstructive pulmonary disease, PCI: percutaneous coronary intervention, LV: left ventricular, NYHA: New York Heart Association functional class, CAD: coronary artery disease, EF: left ventricular ejection fraction, LVEDD: left ventricular end-diastolic dimension, LAD: left atrial dimension, MR: mitral regurgitation, BWMA: left ventricular inferior basal wall motion abnormality, CABG: coronary artery bypass graft, LIMA: left internal mammary artery, TAP: tricuspid annuloplasty, ACC: aortic cross-clamp, CPB: cardiopulmonary bypass, IABP: intra-aortic balloon pump, ICU: intensive care unit.

*Indicates variables entered into logistic regression for propensity score matching.

### Follow-up and outcomes

The clinical follow-up was closed on April 1, 2017. The median follow-up was 59 months (interquartile range, 37–85 months) with a completion rate of 98.4% (429/436) in the overall cohort. During follow-up, 63 patients (14.4%) died, of whom 50 (79.4%) died of a cardiac cause. After adjustment for baseline differences with Cox proportional hazard model analysis, there was no significant difference between MVA and MVR in risks of major adverse cardiac or cerebrovascular events (MACCE: cardiac death, repeat revascularization and myocardial infarction, stroke, subsequent mitral valve surgery, or hospitalization for heart failure), cardiac death, or overall death (for MACCE: *P* = 0.163; for cardiac death: *P* = 0.228; and for overall death: *P* = 0.268) (Table 2).

**Table 2:** Long-term outcomes according to different surgical procedures in the overall population.

	MVA n (%)	MVR: n (%)	Adjusted HR^#^ (95% CI)	P Value
All patients	316	120		
Cardiac death	41(13.0%)	15(12.5%)	1.50(0.78–2.89)	0.228
Overall death	51(16.1%)	18(15.0%)	1.39(0.78–2.50)	0.268
MACCE	88(27.8%)	21(17.5%)	0.69(0.41–1.16)	0.163

HR: hazard ratio, CI: confidence interval, MACCE: major adverse cardiac and cerebrovascular event, MVA: mitral valve annuloplasty, MVR: mitral valve replacement.

^#^Multivariable Cox proportional hazard analysis was used with adjustment for all patient-level variables (Indicated by^*^) in Table 1. The HRs were reported for MVA with MVR as reference.

### Risk factors and prespecifed subgroup analysis

**Figure 1:**
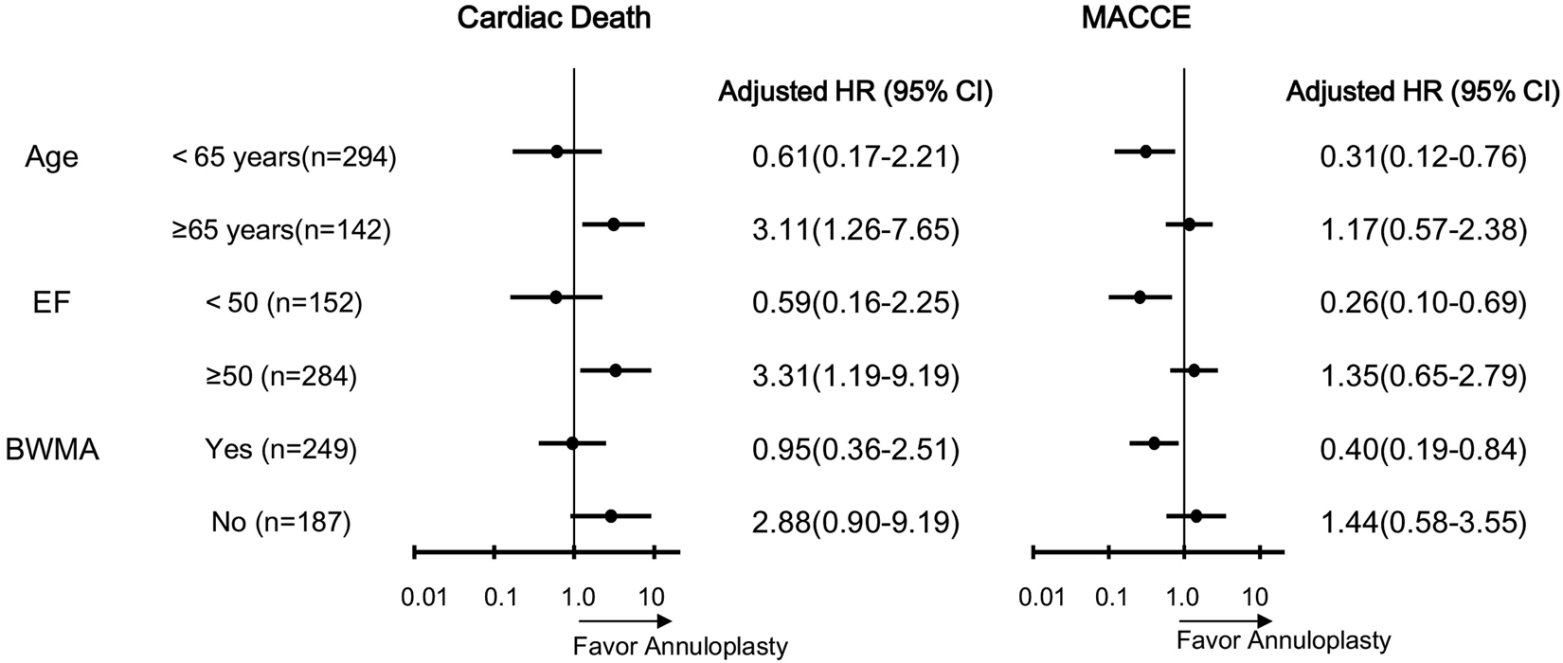
Hazard ratios (HRs) associated with surgical procedures in prespecifed subgroups of patients. Subgroup analyses were performed with the use of Cox proportional hazard analysis with adjustment for all patient-level variables (Indicated by*) in Table 1. The HRs were reported for MVR with MVA as reference. HR: hazard ratio, CI: confdence interval, MACCE: major adverse cardiac and cerebrovascular event, EF: left ventricular ejection fraction, BWMA: left ventricular inferior basal wall motion abnormality.

Cox proportional hazard model analysis showed that both age and preoperative EF were independent predictors of cardiac death at follow-up (for age: HR, 1.04; 95% CI, 1.01–1.07, *P* = 0.011; and for EF: HR, 0.95; 95% CI, 0.93–0.97, *P* < 0.001). Of note, the choice of MVA or MVR was not a significant predictor of late cardiac death (*P* = 0.233) (Table 3). We also assessed the relative surgical procedures effects in subgroups of patients with major high-risk clinical factors. MVA provides better results than MVR in terms of freedom from cardiac death in subgroups of age ≥65years and EF ≥50% (for age: *P* = 0.014; and for EF: *P* = 0.016), whereas MVR was associated with a lower risk of MACCE than MVA in subgroups of age < 65years, EF < 50% and BWMA (for age: *P* = 0.010; for EF: *P* = 0.007, and for BWMA: *P* = 0.016) (Fig. 1).

**Table 3:** Cox proportional hazard analysis for cardiac death at long-term follow-up.

Predictors	Univariable		Multivariable	
	P value	HR (95% CI)	P value	HR (95% CI)
Surgical procedures^*^	0.501	1.23(0.68–2.22)	0.233	
Age	0.029	1.04(1.01–1.07)	0.011	1.04(1.01–1.07)
History of heart failure	0.122	1.52(0.89–2.60)	0.779	
EF	<0.001	0.95(0.93–0.97)	<0.001	0.95(0.93–0.97)
Postoperative IABP	0.060	2.27(0.97–5.32)	0.464	

HR: hazard ratio, CI: confdence interval, EF: left ventricular ejection fraction, IABP: intra-aortic balloon pump.

^*^Indicates mitral valve annuloplasty or replacement.

### Results of propensity score matching analysis

**Figure 2:**
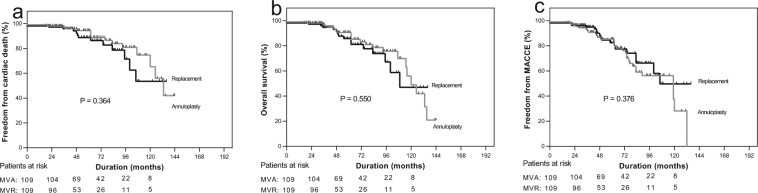
Kaplan-Meier curves for (**a**) freedom from cardiac death (**b**) overall survival and (**c**) freedom from MACCE in 1:1 propensity score-matched annuloplasty group (gray lines) and replacement group (black lines). MVA: mitral valve annuloplasty, MVR: mitral valve replacement; MACCE: major adverse cardiac and cerebrovascular event (cardiac death, repeat revascularization and myocardial infarction, stroke, subsequent mitral valve surgery, or hospitalization for heart failure).

In the propensity score matching analysis, 109 pairs were extracted by 1:1 manner using nearest neighbor matching without replacement. There was no signifcant difference between the two propensity-matched groups with regard to baseline characteristics (Table 1). However, compared with MVA, the aortic cross-clamp time, cardiopulmonary bypass time and the duration of intensive care unit (ICU) for MVR were markedly longer (*P* < 0.05) (Table 1). The incidences of early mortality and postoperative complications (stroke, reoperation for bleeding, application of intra-aortic balloon pump and acute renal failure) were also similar between the two propensity score-matched groups, except for a higher incidence of respiratory complications in the MVR group than in the MVA group (Table 4). During the follow-up, compared with the MVR group, the left ventricle end-diastolic diameter was markedly larger (*P* = 0.019), and the incidence of mitral regurgitation recurrence was significantly higher in the MVA group (*P* < 0.001) (Table 5), however, we observed no significant difference in overall survival, freedom from cardiac death or MACCE between MVA and MVR (Fig. 2, all *P* > 0.05).

**Table 4:** Early clinical outcomes of propensity score-matched patients.

Variables	MVA (n = 109)	MVR (n = 109)	P Value
In-hospital mortality: n (%)	1(0.9)	2(1.8)	0.557
Complications: n (%)	12(11.0)	21(19.3)	0.089
Stroke: n (%)	0(0)	1(1.0)	—
Reoperation for bleeding: n (%)	2(1.8)	4(3.7)	0.403
Postoperative IABP: n (%)	5 (4.6)	4 (3.7)	0.733
Respiratory complication: n (%)	3(2.8)	10(9.2)	0.040
Acute renal failure: n (%)	2 (1.8)	2 (1.8)	>0.999

MVA: mitral valve annuloplasty, MVR: mitral valve replacement, IABP: intra-aortic balloon pump.

**Table 5:** Perioperative and follow-up echocardiographic results of propensity score-matched patients.

Variables	MVA (n = 109)			MVR (n = 109)		
	Preoperative	Postoperative	Follow-up	Preoperative	Postoperative	Follow-up
EF (%)	54.94 ± 10.42	52.92 ± 9.78	54.18 ± 9.56	56.11 ± 10.06	52.71 ± 9.09	52.70 ± 10.07
LVEDD mid-ventricle (mm)	58.04 ± 6.46	50.58 ± 5.55	55.24 ± 6.37	58.43 ± 6.25	51.46 ± 7.34	53.13 ± 6.78^*^
LAD (mm)	43.63 ± 6.60	38.56 ± 5.24	44.32 ± 5.86	43.91 ± 7.11	39.95 ± 5.41	43.50 ± 6.47
MR, n (%)	—	—	63(57.80%)	—	—	3(2.75%)^*^
Moderate	—	—	49(44.95%)	—	—	3(2.75%)
Severe	—	—	14(12.84%)	—	—	0
Periprosthetic leak	—	—	—	—	—	2(1.83%)

MVA: mitral valve annuloplasty, MVR: mitral valve replacement, EF: left ventricular ejection fraction, LVEDD: left ventricular end-diastolic dimension, LAD: left atrial dimension, MR: mitral regurgitation. ^*^P<0.05 versus MVA.

## Discussion

In the present study, the incidences of early mortality and postoperative complications were similar between the two propensity score-matched groups. According to published literatures, compared with MVR, early mortality and complications for MVA fall into 2 categories: no difference and lower incidence. Several recent experiences have failed to detect any substantial difference between the two surgical managements in terms of in-hospital mortality or complications, which are in accordance with our observations^2,9^, whereas several studies showed that mitral valve repair is associated with lower operative mortality^7,8^.

In the present long-term observational study, after adjustment for baseline differences with Cox proportional hazard model and propensity score matching analysis, we observed no significant difference between MVA and MVR in risks of MACCE, cardiac death, or overall death. Follow-up echocardiographic results of propensity score-matched patients showed that, compared with the MVR group, the left ventricle end-diastolic diameter was markedly larger, and the incidence of mitral regurgitation recurrence was significantly higher in the MVA group. The optimal surgical approach to the treatment of severe ischemic mitral regurgitation remains controversial. Published series have provided a wide range of results for long-term outcomes. A multicenter, randomized trial conducted by the Cardiothoracic Surgical Trials Network for severe ischemic mitral regurgitation showed that two-year mortality was 19.0% in the MVA group and 23.2% in the MVR group (*P* = 0.39)^2^. An important study carried out by Lorusso and colleagues showed that eight-year survival was 81.6% ± 2.8% versus 79.6% ± 4.8% in MVA and MVR, respectively (*P* = 0.42)^3^. A recent meta-analysis showed that MVA is associated with higher recurrence of MR in patients with CIMR, and no difference was found regarding survival, NYHA class, and functional indicators^7^. Cohn and colleagues^10^ reported a 5-year survival of 56% and 91.5% in MVA and MVR, respectively, whereas a meta-analysis showed that the relative long-term risk of death was 35% higher in the MVR group than in the repair group^11^.

Such different conclusions might have derived from the heterogeneity of patient cohorts. Therefore, in the present study, we included only patients undergoing MVA or MVR with complete myocardial revascularization. We also excluded patients with congenital valvular heart disease, rheumatic valvular disease, infective endocarditis, presence of aortic valve regurgitation or stenosis, or receiving other procedures. Moreover, to minimize the effects of confounding variables, a propensity score model was constructed.

An important study showed that both older age and lower preoperative left ventricular EF were independent predictors of cardiac death at follow-up^12^, which was in consistent with the present study. Considering MVA and MVR have different characteristics in terms of operative mortality, long-term correction of mitral regurgitation and long-term thromboembolism, mitral procedure selection should be individualized and depend on major high-risk clinical factors. As a result, we assessed the effects of both surgical procedures in subgroups of patients with major high-risk clinical factors. In this study, prespecifed subgroup analysis showed that, during long-term follow-up, MVR was associated with a lower risk of MACCE than MVA in subgroups of age < 65years, EF < 50% and BWMA.

Compared with MVA, MVR provides a considerably more durable correction of MR^2,8^, which may have a beneficial effect on long-term outcomes. However, this effect must be weighed against any potential adverse consequences of a prosthetic valve, such as long-term thromboembolism, endocarditis, and structural valve deterioration^2^. Recent studies have showed that, compared with MVR, MVA provides worse long-term MR correction with risks of adverse left ventricular remodeling, readmission and poor survival^2^. Multiple studies have attempted to develop predictive models of MR recurrence based on preoperative parameters^13–16^. Some studies pointed out that poor LV function and preoperative basal aneurysm/dyskinesis were key predictive factors of MR recurrence^13,17^. BWMA reflects severe LV ischemic remodeling including papillary muscle displacement, and leaflet tethering, all of which influence CIMR. Kron and colleagues concluded that the presence of preoperative basal aneurysm/dyskinesis was strongly associated with MR recurrence, and the mechanism for MR recurrence was largely mitral valve leaflet tethering^16^.

There are still some limitations. First, this study reports retrospective data from a single center and is subject to all the limitations inherent to this design. Second, the small study sample might have led to type II statistical errors. An appropriately powered, randomized, controlled trial evaluating the optimal management of CIMR would be useful to confirm our results. Third, pre-, intra-, and postoperative information about the exact mechanisms and characteristics of MR were not available in all patients.

In the current study, though MVR was more favorable to ventricular remodeling than MVA, they provided comparable results in terms of overall survival, freedom from cardiac death and avoidance of MACCE at follow-up. Mitral procedure selection should be individualized and depend on major high-risk clinical factors. With age ≥65years or EF ≥50%, MVA is the optimal management of severe CIMR, whereas MVR would be a better alternative under the condition of age < 65 years, EF < 50% or BWMA.

## Methods

### Patients and study design

This study was approved by the Human Research Ethics Committee of the Fuwai Hospital and was performed in accordance with the Declaration of Helsinki and the approved guidelines. Oral informed consent was obtained from all of the patients via a telephone questionnaire. CIMR was defined by coronary angiographic and echocardiographic findings according to accepted criteria, i.e., 1) Mitral regurgitation (MR) occurring more than 16 days after myocardial infarction; 2) type I/IIIb leaflet dysfunction following Carpentier’s classification; and 3) 70% or greater stenosis of at least one coronary artery, with wall motion abnormalities of the corresponding left ventricular segment^3^.

Between January 2002 and December 2014, a total of 1066 patients with CIMR were hospitalized for undergoing coronary artery bypass grafting (CABG) combined with MVA or MVR. From the initial cohort, 630 patients were excluded for various reasons^3^, i.e., 1) Preoperative MR ≤2+, congenital valvular heart disease, rheumatic or degenerative valvular disease, infective endocarditis, presence of aortic valve regurgitation or stenosis, emergency surgery, or repeat operation; or 2) performance of other procedures, such as left ventricular reconstruction/reshaping, or procedures other than mitral ring annuloplasty for the treatment. Thus, the final study cohort comprised 436 patients: 316 patients (72.5%) underwent MVA whereas 120 (27.5%) underwent MVR. 7 (1.6%; 5 in MVA group and 2 in MVR group) patients were lost to follow up.

Baseline patient characteristics, echocardiography data, operative data, and surgical techniques were collected from the division of cardiovascular surgery’s database and individual medical records. Patients were followed up through internet or telephone interviews and the outpatient department records. All collected data were sent to a core lab (State Key Laboratory of Cardiovascular Disease, Beijing, China) for analysis.

### Surgical technique

All surgical procedures were performed with standard bypass techniques through median sternotomy by senior surgeons with special interest in mitral valve surgery. The decision to perform MVA or MVR was at the surgeon’s discretion. Downsizing ring annuloplasty (2 sizes) was used in all patients subjected to MVA^3,18^. Subvalvular apparatus were preserved for MVR whenever possible (94/120, 78.3%), including posterior leaflet preservation, posterior and partial anterior leaflet preservation, both leaflets preservation. The decision to perform which kind of procedure was at the surgeon’s discretion according to the condition. The posterior leaflet preservation was performed in 58 patients undergoing MVR. In 8 of patients undergoing MVR, the middle portion of the anterior leaflet was resected and the remaining leaflet tissue was plicated with the individual valve sutures. In 28 of the patients undergoing MVR, the anterior leaflet of the valve was partly or completely detached from the mitral annulus and divided in the middle at the 12 o’clock position, and the leftward portion of the anterior leaftlet was plicated to the anterolateral commissure with a pledgetted 4–0 polypropylene suture. The rightward portion of the anterior mitral leaflet was similarly plicated to the posteromedial commissure. Complete revascularization was achieved in all patients with arterial conduits or saphenous vein grafts. All patients received the same perioperative care and medical therapy according to guidelines.

### Echocardiography

Two-dimensional and Doppler transthoracic echocardiography examinations were performed before operation and at predischarge for all patients. MR was classified as mild (grade 1+), moderate (grade 2+), or severe (grades 3+ and 4+)^19^. Left ventricular (LV) inferior basal wall motion abnormality (BWMA) includes hypokinesia, dyskinesis and an aneurysm. Echocardiographic criteria for aneurysm were evidence of thinning and localized LV dilation or distortion. Dyskinesis was the presence of outward displacement of the LV wall during systole^20,21^.

### Statistical analysis

All statistical analyses were performed by SAS software version 9.2 (SAS Institute), SPSS version 20 (IBM SPSS Inc., Chicago, IL) and Graph Pad Prism release 5 (Graph Pad Software Inc, La Jolla, Calif) statistical packages. All reported *P* values are 2 sided, and values of *P* < 0.05 were considered to indicate statistical signifciance. Values are expressed as a mean ± SD, median with range, or proportion. Comparisons between the two groups were performed using the chi-square test or Fisher’s exact test for categoric variables and *t* test for continuous variables. The Wilcoxon rank sum test was used for variables not normally distributed. A stepwise multivariable Cox proportional hazards model was developed to determine the independent risk factors. Variables with a *P* value less than 0.15 in the univariate analyses were entered into multivariable models. Differences in risk-adjusted, long-term rates of study outcomes among patients underwent different surgical procedures were assessed by use of multivariable Cox proportional hazards regression with adjustment for all patient-level variables in Table 1. Cumulative event rates were calculated using a Kaplan-Meier method, and different event curves of outcomes were compared using a Log-Rank test. Surgical procedures related differences in long-term outcomes were also analyzed in high-risk clinical subgroups.

To reduce the impact of treatment selection bias and potential confounding in the observational study, we performed rigorous adjustment for baseline differences by use of propensity score matching^22^. A propensity score representing the probability of having MVR as opposed to MVA was calculated for each patient by using a nonparsimonious multivariable logistic regression model. Variables used in the model are shown in Table 1. Pairs of patients with MVA and MVR were matched using calipers of width 0.2 standard deviations of the logit of the propensity score^23^. Model discrimination was assessed with C statistics, and model calibration was assessed with Hosmer-Lemeshow statistics. Finally, 109 pairs of patients were matched to obtain risk-adjusted outcome comparisons between the two groups.

## References

[1] Acker MA (2014). Mitral-Valve Repair versus Replacement for Severe Ischemic Mitral Regurgitation. New England Journal of Medicine.

[2] Goldstein D (2016). Two-Year Outcomes of Surgical Treatment of Severe Ischemic Mitral Regurgitation. N Engl J Med.

[3] Lorusso, R. *et al*. Mitral valve repair or replacement for ischemic mitral regurgitation? The Italian Study on the Treatment of Ischemic Mitral Regurgitation (ISTIMIR). *J Thorac Cardiovasc Surg* **145**, 128–139, discussion 137–128, 10.1016/j.jtcvs.2012.09.042 (2013). 10.1016/j.jtcvs.2012.09.042 23127376

[4] Di Salvo TG, Acker MA, Dec GW, Byrne JG (2010). Mitral valve surgery in advanced heart failure. J Am Coll Cardiol.

[5] Perrault LP (2012). Optimal surgical management of severe ischemic mitral regurgitation: to repair or to replace?. J Thorac Cardiovasc Surg.

[6] Nishimura RA (2017). AHA/ACC Focused Update of the 2014 AHA/ACC Guideline for the Management of Patients With Valvular Heart Disease: A Report of the American College of Cardiology/American Heart Association Task Force on Clinical Practice Guidelines. J Am Coll Cardiol.

[7] Wang J, Gu C, Gao M, Yu W, Yu Y (2015). Mitral valve replacement therapy causes higher 30-day postoperative mortality than mitral valvuloplasty in patients with severe ischemic mitral regurgitation: A meta-analysis of 12 studies. Int J Cardiol.

[8] Dayan V, Soca G, Cura L, Mestres CA (2014). Similar survival after mitral valve replacement or repair for ischemic mitral regurgitation: a meta-analysis. The Annals of thoracic surgery.

[9] Maltais S (2011). Mitral regurgitation surgery in patients with ischemic cardiomyopathy and ischemic mitral regurgitation: factors that influence survival. J Thorac Cardiovasc Surg.

[10] Cohn LH (1995). The effect of pathophysiology on the surgical treatment of ischemic mitral regurgitation: operative and late risks of repair versus replacement. European journal of cardio-thoracic surgery: official journal of the European Association for Cardio-thoracic Surgery.

[11] Vassileva CM, Boley T, Markwell S, Hazelrigg S (2011). Meta-analysis of short-term and long-term survival following repair versus replacement for ischemic mitral regurgitation. European journal of cardio-thoracic surgery: official journal of the European Association for Cardio-thoracic Surgery.

[12] Magne J (2009). Mitral repair versus replacement for ischemic mitral regurgitation: comparison of short-term and long-term survival. Circulation.

[13] Gelsomino S (2008). Five-year echocardiographic results of combined undersized mitral ring annuloplasty and coronary artery bypass grafting for chronic ischaemic mitral regurgitation. European heart journal.

[14] Gelsomino S (2011). Impact of preoperative anterior leaflet tethering on the recurrence of ischemic mitral regurgitation and the lack of left ventricular reverse remodeling after restrictive annuloplasty. Journal of the American Society of Echocardiography: official publication of the American Society of Echocardiography.

[15] Digiammarco G (2007). Recurrence of functional mitral regurgitation in patients with dilated cardiomyopathy undergoing mitral valve repair: how to predict it. Interactive cardiovascular and thoracic surgery.

[16] Kron IL (2015). Predicting recurrent mitral regurgitation after mitral valve repair for severe ischemic mitral regurgitation. J Thorac Cardiovasc Surg.

[17] Shiota M, Gillinov AM, Takasaki K, Fukuda S, Shiota T (2011). Recurrent mitral regurgitation late after annuloplasty for ischemic mitral regurgitation. Echocardiography.

[18] Vahanian A (2012). Guidelines on the management of valvular heart disease (version 2012): the Joint Task Force on the Management of Valvular Heart Disease of the European Society of Cardiology (ESC) and the European Association for Cardio-Thoracic Surgery (EACTS). European journal of cardio-thoracic surgery: official journal of the European Association for Cardio-thoracic Surgery.

[19] Zoghbi WA (2003). Recommendations for evaluation of the severity of native valvular regurgitation with two-dimensional and Doppler echocardiography. Journal of the American Society of Echocardiography: official publication of the American Society of Echocardiography.

[20] Weyman AE, Peskoe SM, Williams ES, Dillon JC, Feigenbaum H (1976). Detection of left ventricular aneurysms by cross-sectional echocardiography. Circulation.

[21] Lebeau R (2003). A new tool for estimating left ventricular ejection fraction derived from wall motion score index. The Canadian journal of cardiology.

[22] Li F, Zaslavsky AM, Landrum MB (2013). Propensity score weighting with multilevel data. Statistics in medicine.

[23] Austin PC (2011). Optimal caliper widths for propensity-score matching when estimating differences in means and differences in proportions in observational studies. Pharmaceutical statistics.

